# Sustainable logistics development strategy based on SWOT and analytic hierarchy process

**DOI:** 10.1371/journal.pone.0312560

**Published:** 2024-10-23

**Authors:** Changgang Xiao

**Affiliations:** School of Economics and Management, Qilu Normal University, Jinan, China; Gonbad Kavous University, ISLAMIC REPUBLIC OF IRAN

## Abstract

Due to the implementation of sustainable green development strategy, the study uses the competitive situation method and analytical hierarchy process of logistics enterprises to conduct structural analysis and weight calculation of factor indicators for their development strategies. Afterwards, the exponential smoothing prediction method was used to weight the logistics data. Moreover, through comparative analysis of the indicator evaluation system, the average weights of the advantageous factors of logistics enterprises were found to be 0.0232, 0.1102, 0.0522, and 0.0468, respectively. The energy consumption, transportation volume, and turnover of logistics transportation increased by 68.9% and 52.3% in 2022, respectively, which had a significant impact on the sustainable development of the logistics industry. It has been demonstrated that a sustainable logistics development strategy based on the enterprise competitive situation method and the analytic hierarchy process is both feasible and superior. Furthermore, this strategy has the potential to enhance the environmental benefits and green development of the economy and society.

## 1. Introduction

The excessively pursued high-energy consumption development model hinders the development of ecological balance and undermines it. Therefore, to protect the ecological environment and maintain ecological balance, the socio-economic development concept is green, harmonious, coordinated and efficient. While adapting to the rapid development of the social economy, green and low-carbon also promote the high-quality development of social industries [1]. At present, the logistics industry becomes an important industry support for national economic development. Under the green Sustainable Development (SD) theory, the logistics industry takes Sustainable Logistics Development (SLD) as the direction and goal of its future development [2]. The strategic analysis of enterprise development is related to different industrial tasks and development directions. The multi-scheme optimization decision analysis method is usually used to calculate the weights of target factors at different levels to quantitatively analyze the influencing factors. The commonly used factor decision analysis methods include multi-objective decision-making, deterministic and uncertain risk decision-making, simple weighting method, Analytic Hierarchy Process (AHP), and network analysis method. These methods quantitatively analyze the factor weights and data in different indicator divisions to help decision-makers formulate development plans. A development strategy analysis focusing on industry enterprises is conducted in order to gain a comprehensive understanding of the characteristics of the industry itself and of the external factors that exert influence. The analysis considers the development strategy and direction of enterprises from four dimensions: advantages, disadvantages, opportunities, and challenges. Accordingly, the Strengths, Weaknesses, Opportunities, and Threats (SWOT) analysis method is a valuable tool for industry and enterprise development strategies, aligning well with the inherent strengths and challenges of development. Furthermore, it offers a robust decision-making framework for achieving SD goals. The logistics industry involves various industrial structures and environments. The modern transportation industry accounts for a relatively large proportion of the entire logistics system. The energy resources it consumes are relatively extensive and have a large capacity, leading to environmental pollution and ecological balance disruption [3]. In addition, the use of packaging materials in logistics systems also belongs to a consumption-oriented industry. Recycling packaging materials can also cause waste of resources and environmental damage to a certain extent. Therefore, SLD needs to utilize advanced technology and environmental protection to save and recycle resources and the environment to meet the reasonable commercial activities of commodity consumption [4,5]. Based on this, the study aims to construct a SLD strategy method using the SWOT and AHP analysis methods to improve the efficient management of the logistics industry and promote harmonious socio-economic development.

## 2. Literature review

In response to the current status of development of the entire logistics system, there are varying degrees of development trends evident in each link and industrial structure. Presently, logistics enterprises are pursuing strategies that facilitate supply chain optimization and conducting competitive analyses to achieve high-quality development tasks. In recent years, many scholars have conducted extensive research on different logistics networks, management systems, and green logistics development plans. Liu et al. [6] used a two-stage mixed integer linear programming model to coordinate the transportation routes between trains and obtain the optimal logistics plan for the logistics scheduling problem in energy systems. Ultimately, the energy penetration rate in the Northeast region increased to 95.3%, reducing the peak energy load and optimizing railway transportation routes. Hu et al. [7] proposed the use of multi-objective collaborative evolution optimization method to optimize the subway passenger and freight operation system for underground logistics system’s network planning. It achieved the optimal configuration scheme for urban automated transportation. Hu et al. [8] proposed to construct a dual objective dynamic programming model in the underground logistics system network to improve its delivery demand for urban garbage and waste collection and package transportation. This saved a significant amount of environmental governance costs and promoted sustainable economic development. In energy scheduling and underground logistics network operation, logistics configuration is adopted to optimize the operation structure, thereby improving work efficiency and reducing the loss of energy resources. In a recent study, Wang et al. [9] proposed the use of a SWOT method to evaluate the factors affecting renewable energy technology in Pakistan’s renewable energy resource strategy. This approach was combined with a fuzzy AHP to analyze government factors, with the goal of alleviating the energy crisis and improving national energy security. Solangi et al. [10] proposed a strategy for evaluating sustainable energy planning in Pakistan by combining SWOT method, AHP method, and fuzzy ranking technique. The robustness of the proposed solution was verified through sensitivity analysis, providing a systematic evaluation method for sustainable energy planning strategies and policies in Pakistan.

However, in the development of the logistics industry and enterprises, vehicle and robot delivery issues play an important role. Gan et al. [11] proposed using Pearson analysis to analyze the evolutionary characteristics of logistics efficiency in Jiangxi Province to promote the transformation of economic development mode to evaluate the efficiency of green logistics. Li and Li [12] proposed an immune genetic algorithm to optimize logistics scheduling for foreign trade service logistics and warehousing systems. A biological immune system was combined to improve the clone immune genetic algorithm, thereby improving the accuracy and quality of the optimal solution for logistics scheduling. Alsudani et al. [13] proposed using the Internet of Things to optimize the configuration of enterprise management systems for intelligent logistics to improve the automation of enterprise logistics management. Tao et al. [14] proposed a multi-vehicle routing method using order splitting and allocation for the vehicle delivery in e-commerce systems. This method used variable neighborhood search to improve the accuracy of heuristic algorithms, thereby providing efficient path planning for vehicle delivery. Shimizu et al. [15] proposed the use of dynamic wireless capability transmission systems to improve the performance of in vehicle logistics robots, reduce losses, and enhance the automation of vehicle loading. In other fields, logistics activities also effectively reduced energy consumption and promoted economic and social development. Nalbur and Yavas [16] proposed using system dynamics and discrete event simulation to study reverse logistics applications for green logistics activities in electric buses. This reduced the carbon emissions of electric buses by 22% to achieve the strategy of green logistics activities. Guo and Li [17] proposed using an expression optimization model to address the carbon emissions in the regional logistics industry. This study used particle swarm optimization algorithms to improve model prediction accuracy and effectively manage logistics carbon emissions. Table 1 summarizes the relevant work.

**Table 1:** Summary table of relevant literature.

Reference	Research contents	Research meaning
Liu et al. [6]	Use a two-stage mixed integer linear programming model to coordinate the transportation routes between trains.	Reduced peak energy load and optimized railway transportation routes.
Hu et al. [7]	Using multi-objective collaborative evolutionary optimization method to optimize the subway passenger and freight operation system.	The optimal configuration scheme for achieving urban automated transportation.
Hu et al. [8]	Build a dual objective dynamic programming model in the underground logistics system network.	Saved a significant amount of environmental governance costs and promoted sustainable economic development.
Wang et al. [9]	Use SWOT method and fuzzy analytic hierarchy process to evaluate the factors affecting renewable energy technologies.	Relieve the energy crisis and enhance national energy security.
Solangi et al. [10]	Combining SWOT, AHP, and fuzzy ranking techniques to evaluate strategies for sustainable energy planning.	Provided a systematic evaluation method for sustainable energy planning strategies and policies in Pakistan.
Gan et al. [11]	Using Pearson analysis to analyze the evolutionary characteristics of logistics efficiency in Jiangxi Province.	Promote the transformation of economic development mode.
Li and Li [12]	Propose a biological immune system to improve the clone immune genetic algorithm for optimizing logistics scheduling problems.	Improve the accuracy and quality of the optimal solution for logistics scheduling.
Alsudani et al. [13]	Optimize and configure enterprise management systems using IoT technology.	Improve the automation level of enterprise logistics management.
Tao et al. [14]	Using the multi vehicle routing method of order splitting and allocation, variable neighborhood search can improve the accuracy of heuristic algorithms.	Provide efficient path planning for vehicle delivery.
Shimizu et al. [15]	Using a dynamic wireless capability transmission system to improve the performance of robots.	Enhanced the automation level of car loading.
Nalbur and Yavas [16]	Using system dynamics and discrete event simulation to study reverse logistics applications.	Reduced carbon emissions from electric buses.
Guo and Li [17]	Use particle swarm optimization algorithm to construct expression optimization model.	Effectively manage logistics carbon emissions.

In summary, current research has explored the operation of logistics networks and activities in various directions, using optimization algorithms to improve the accuracy of logistics allocation in route planning and transportation configuration in various fields. Nevertheless, there is a dearth of detailed strategic analysis pertaining to the overarching logistics industry and the trajectory of enterprise development. It is imperative to enhance the managerial capabilities of enterprises in accordance with their specific developmental requirements and the prevailing circumstances. The current research results cannot meet the current situation analysis and strategic improvement of specific logistics enterprises. Therefore, the study adopts the SWOT method to design the structure of the enterprise’s current situation and influencing factors, and innovatively uses the exponential smoothing prediction model to quantitatively analyze the key strategic factors. In conclusion, the AHP method is integrated into the construction of an SLD strategy based on SWOT-AHP. This approach not only offers an efficient and environmentally sustainable framework for logistics enterprises, but also provides technical insights that can inform the development of alternative logistics configuration schemes.

## 3. Methods and materials

In the logistics industry and the developing strategic goals of enterprises, the study adopts the enterprise competitive situation analysis method to analyze the four indicators of logistics development. Combined with AHP, the weights of key factors are calculated to determine the SLD plan.

### 3.1 SWOT status of sustainable logistics development strategy

**Fig 1:**
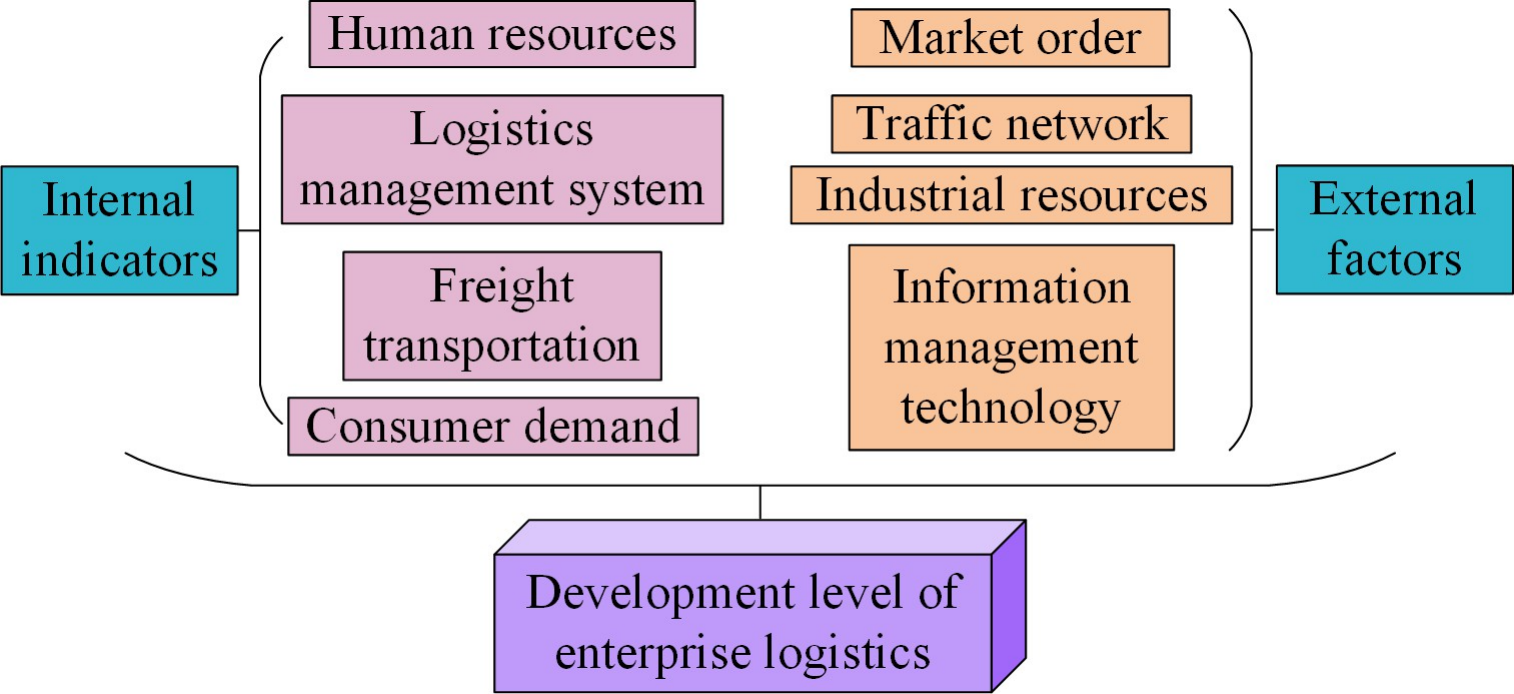
Indicators and structural diagram of regional logistics development.

The development of the logistics industry is influenced by many factors. The interconnection between various factors makes the internal and external factors of logistics development more complex. In the development of the logistics industry, taking the influencing indicators and factors of regional logistics development as an example, Fig 1 shows the structure of logistics development.

**Fig 2:**
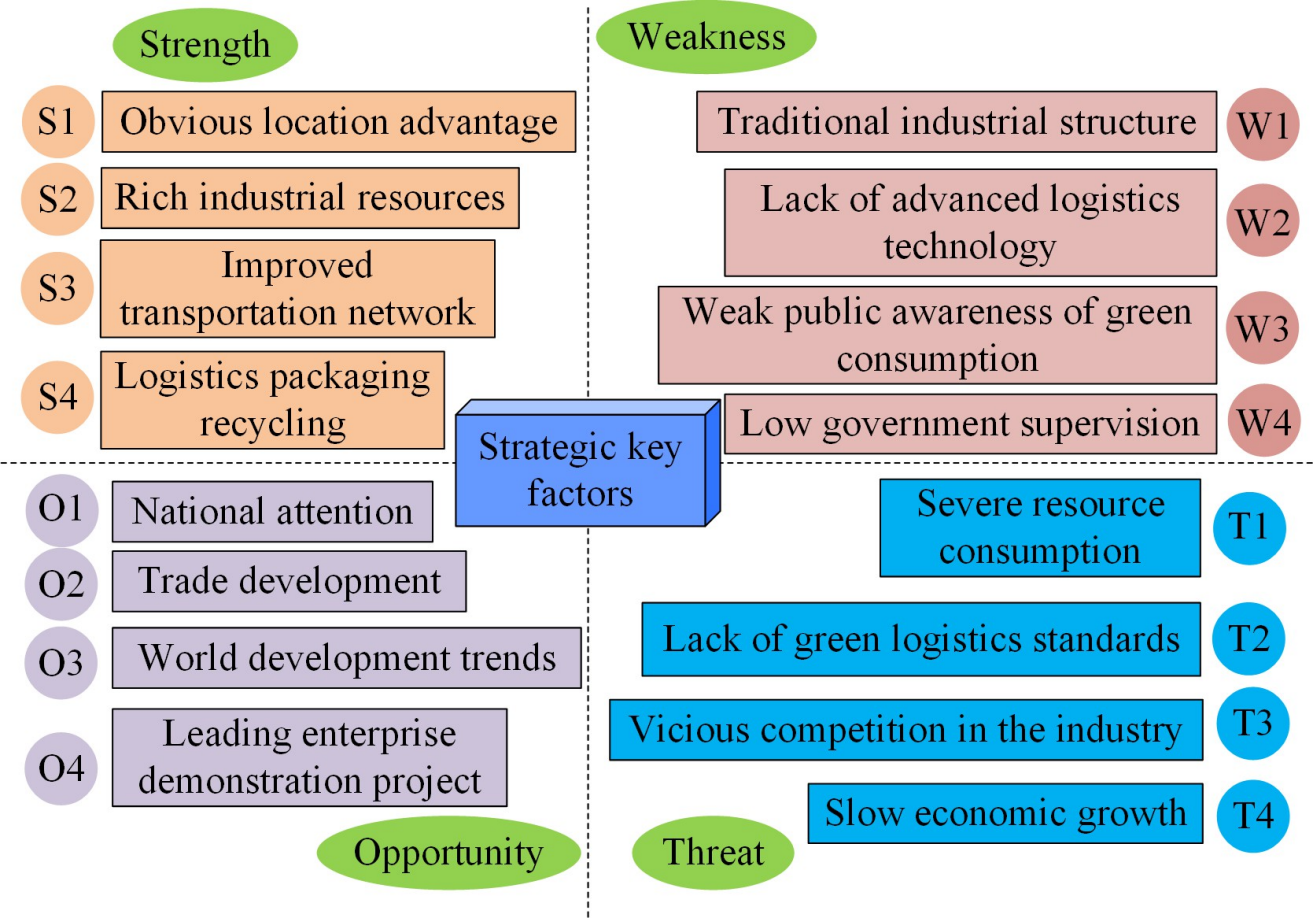
SWOT quantitative analysis model for sustainable logistics development.

From Fig 1, the indicators of regional logistics development are divided into internal factors of enterprises and external economic factors. The internal human resources, management systems, and cargo transportation volume are directly related to the normal operation of the logistics industry. It can be posited that external transportation networks, policy support, and modern advanced information management technologies exert a degree of influence on the development of the logistics economy. Therefore, in the face of complex factor indicators, the study conducts a qualitative analysis of the current SLD using SWTO. Four key factors are extracted from the experiment and combined with expert scoring results to construct a SWOT quantitative analysis model [18] in Fig 2.

From Fig 2, the SWOT quantitative model of SLD mainly includes four strategic key factors. Advantages include location, resources, transportation, and packaging materials. Disadvantages include outdated industrial structure, lack of information technology, government regulation, and talent introduction. Opportunities include the emphasis on national policies, the promotion of international trade, world logistics, and leading enterprises. Threats include resource consumption, green standards, industry competition, and overall economic growth, all of which exert development pressure on the SD of the logistics industry. The above SWOT factors are determined based on the current development status and SD goals of the logistics industry. The quantitative analysis of the strengths, weaknesses, opportunities, and challenges of logistics enterprises is conducted through the examination of development factors, including resources, policies, transportation, and markets. This approach is universally applicable to the SD tasks of logistics enterprises. Afterwards, expert opinions are used to score the factors and establish a judgment matrix for key factors. AHP is then used to calculate the corresponding weights, completing the consistency test of its indicators and providing a multi-level analysis method for the strategic plan of SLD.

### 3.2 Construction of a scoring system for key factors in the development of green logistics

By constructing the SWOT quantitative model of SLD, this study combines expert rating opinions and AHP to establish a mathematical expression matrix for the relative importance of pairwise factors among four systems. As the importance of each indicator in the matrix is reflected in its vector of importance to the overall indicators, and the relationship between the ranking vectors of the indicators is used as the eigenvalue of the judgment matrix, it can be observed that the normalized eigenvalue is capable of representing the weight vector of the factor indicators. Specifically, the eigenvalue method is used to calculate the factor weights and perform consistency checks on the indicators. Firstly, the product of all factors in each row of a judgment matrix *A* is calculated in Eq (1).


$$X_{i}=\prod_{j=1}^{n}a_{ij}(i,j=1,2,3,\ldots ,n)$$ (1)

In Eq (1), *i* represents the row of the judgment matrix. *j* is the column. *a*_*ij*_ represents the impact of elements in row *i* and column *j* on the previous level indicator. *X*_*i*_ means a product calculation. The *n* root of *X*_*i*_ was calculated and represented by Eq (2).


$${\bar{V}}_{i}=\sqrt[{n}]{X_{i}}$$ (2)

In Eq (2), ${\bar{V}}_{i}$ represents the *n* root result of *X*_*i*_, which is the vector, and *i*,*j* = 1,2,3,…,*n*. The vectors are normalized and represented by Eq (3).


$$V_{i}=\frac{{\bar{V}}_{i}}{\sum_{i=1}^{n}{\bar{V}}_{i}}$$ (3)

In Eq (3), *V*_*i*_ represents the sorting weight obtained through normalization. Afterwards, the maximum eigenvalue root can be calculated, represented by Eq (4).


$$\gamma_{\max}=\sum_{i=1}^{n}\frac{{\left(A•V\right)}_{i}}{V_{i}}$$ (4)

In Eq (4), *γ*_max_ represents the maximum value of the eigenvalue root. *V* is the eigenvector of the judgment matrix *A*. (*A*•*V*)_*i*_ is the *i*th element of *A*•*V*. Finally, the Consistency Indicators (CI) of the judgment matrix are calculated, represented by Eq (5).


$$CI=\frac{\gamma_{\max}-n}{n-1}$$ (5)

In Eq (5), *n* represents a specific element. When CI is small, the consistency of the judgment matrix is good. Afterwards, the Random Consistency Average Index (RI) of AHP is combined and used as a constant value parameter. The Consistency Random (CR) is calculated and represented by Eq (6).


$$CR=\frac{CI}{RI}$$ (6)

When it is below 0.1, the consistency test of the judgment matrix is qualified. It also indicates that the expert opinion agrees with the relative importance of various indicators in the system.

### 3.3 Establishment of SWOT strategic structure for logistics enterprises

Based on the weights and weighted scores of strategic key factors, a four level factor evaluation table is established. The SWOT strategic structure is constructed based on the total strength of strategic factors [19], represented by Eq (7).


$$\{\begin{array}{c} S=\sum \frac{S_{a}}{n_{a}}\\ W=\sum \frac{W_{b}}{n_{b}}\\ O=\sum \frac{O_{c}}{n_{c}}\\ T=\sum \frac{T_{d}}{n_{d}} \end{array}$$ (7)

**Fig 3:**
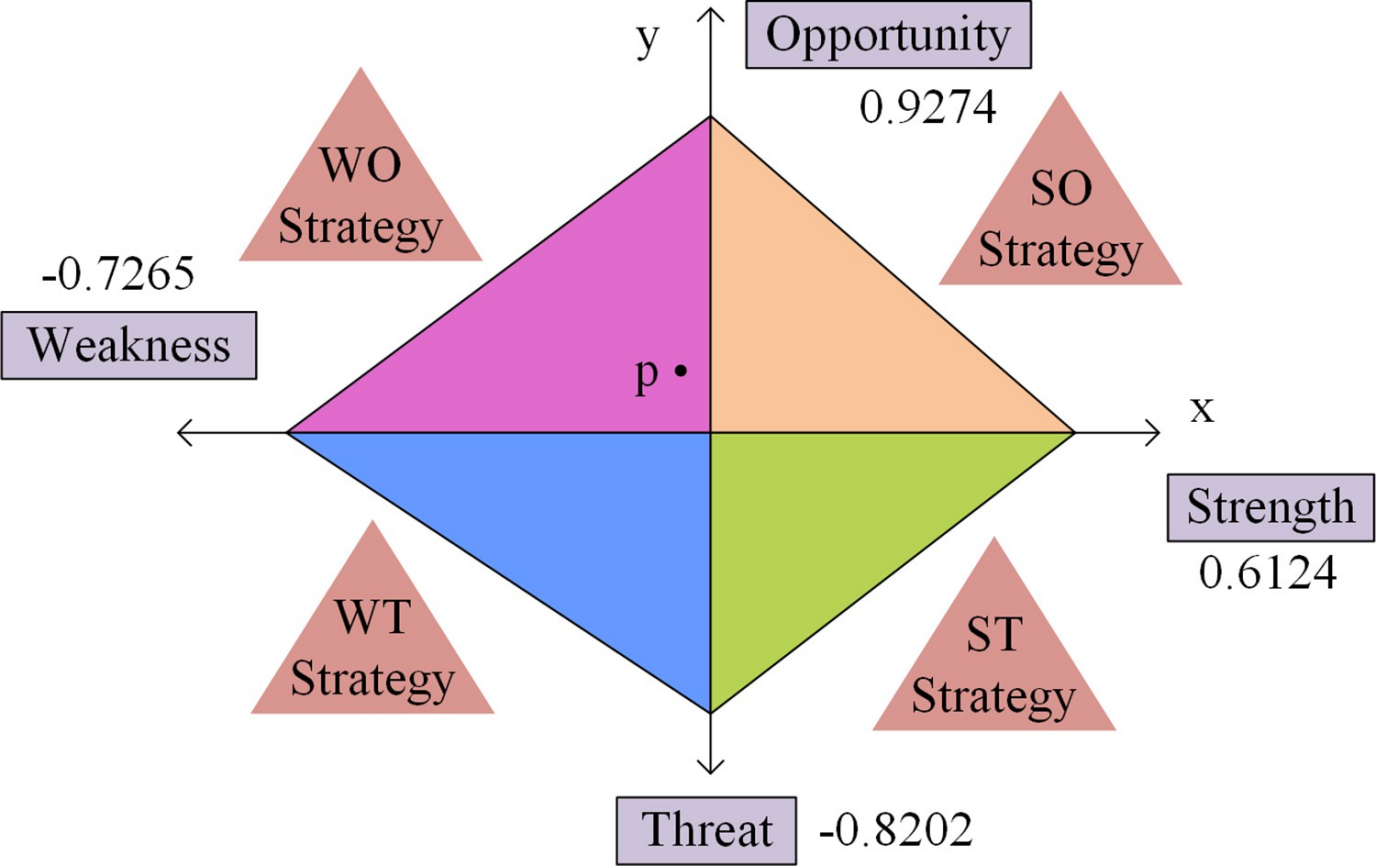
Schematic diagram of SWOT strategic structure.

In Eq (7), *S*, *W*, *O*, and *T* represent the four levels of SWOT, respectively. *n* is the quantity of elements in the corresponding matrix. *a*, *b*, *c*, and *d* represent the quantity of factors that affect the strength of SWOT, respectively. Fig 3 shows the SWOT strategy obtained through factor scoring and factor weighting.

From Fig 3, the overall strength of the four factors in the SWOT strategic structure is inconsistent. However, the triangular area formed determines the priority order of future logistics SD strategies. The coordinate of the development focus is represented by Eq (8).


$$p(x,y)=(\sum \frac{X_{i}}{4},\sum \frac{Y_{i}}{4})$$ (8)

In Eq (8), $\sum \frac{X_{i}}{4}$ represents the average sum of the horizontal coordinates of the four strategic forces. $\sum \frac{Y_{i}}{4}$ is the average sum of the vertical coordinates. The azimuth of *p*(*x*,*y*)’s development strategy is represented by Eq (9).


$$\tan\;\varphi =\frac{y}{x}$$ (9)

In Eq (9), *φ* is the azimuth interval of point *p*, and 0<*φ*<2*π*. The determination of strategic azimuth can determine the direction of SLD. The specific types of development strategies also require a strategic intensity coefficient for classification. The calculation of strategic intensity coefficient is related to the positive and negative intensities of the four strategic angles, as represented by Eq (10).


$$\{\begin{array}{c} P=O\times S\\ Q=T\times W \end{array}$$ (10)

In Eq (10), *P* and *Q* represent the positive and negative intensity operations of strategic development, respectively. Therefore, the coefficient of strategic intensity is represented by Eq (11).


$$\lambda =\frac{P}{P+Q}$$ (11)

In Eq (11), *λ* represents the strategic intensity coefficient. A strategic intensity coefficient below 0.5 indicates that a relatively conservative development strategy is chosen in the strategic areas of disadvantage and opportunity. The environmental awareness of consumers in the disadvantaged strategy also affects the development of sustainable logistics. The recycling of packaging materials can reduce inventory volume by 80%, logistics transportation costs by 75%, and even contribute to the construction of a circular economy system [20].

### 3.4 Calculation method for future sustainable development strategy of logistics industry

**Fig 4:**
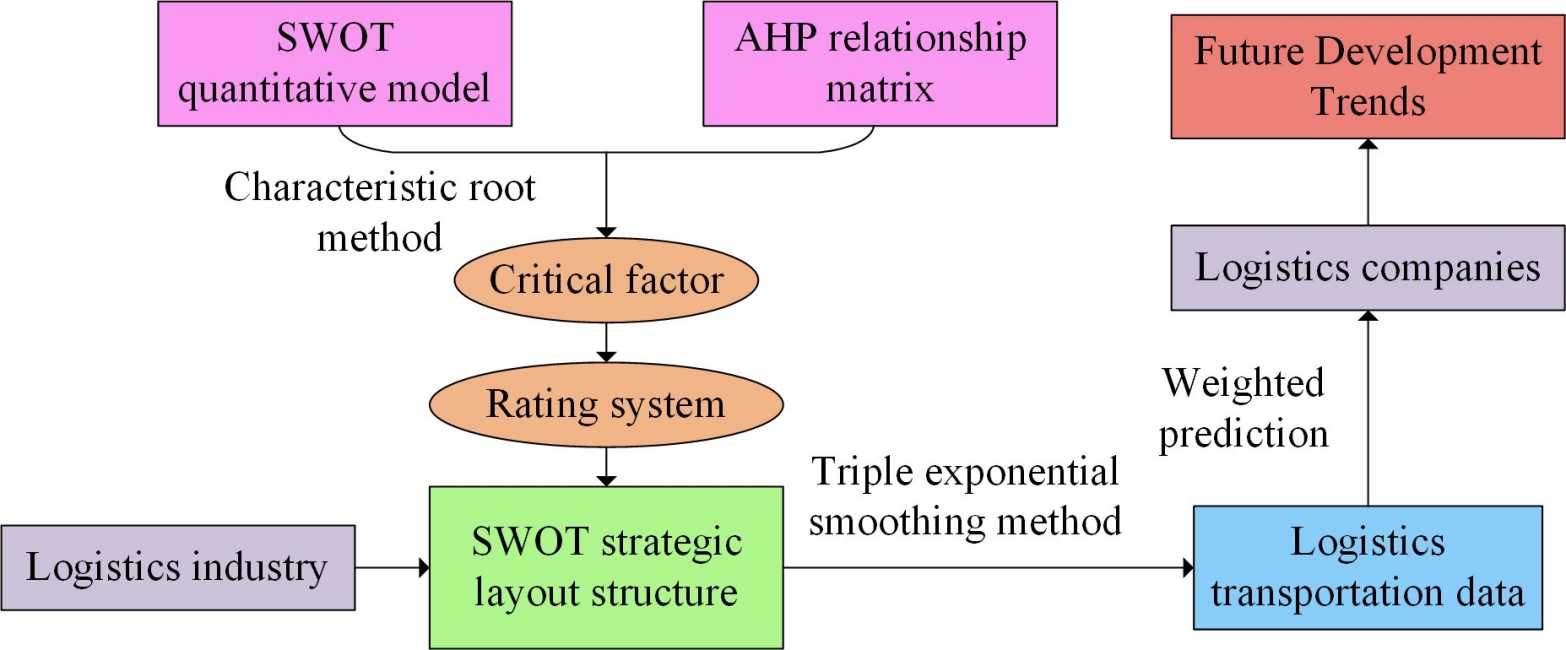
Schematic diagram of sustainable logistics development strategy calculation.

In accordance with the SWOT strategic structure of the logistics industry and the scoring system of key factors, this study calculates the relevant factors and future development trends, as illustrated in Fig 4.

In Fig 4, the SWOT quantitative model and AHP mathematical matrix for SLD have been constructed. Moreover, the key factors have been weighted using the feature method to establish a factor evaluation system. A SWOT strategic structure is constructed by combining strategic factors. The SLD, however, relies on transportation and its quantity. Furthermore, the indicators influencing the logistics industry have practical significance. The simultaneous triple exponential smoothing method can be used to calculate logistics transportation volume data, which provides feasible data for the future development trend of enterprises. Usually, the logistics transportation volume of large enterprises can reach 5 million tons to 40 million tons, which is related to the level of urban development and geographical location [21]. Therefore, the study adopts a triple exponential smoothing prediction method to calculate the future data of logistics transportation volume, and then weights the time series for prediction. Firstly, the initial value of transportation volume is determined, represented by Eq (12).


$$Z_{0}^{\left(1\right)}=Z_{0}^{\left(2\right)}=Z_{0}^{\left(3\right)}=\frac{M_{1}+M_{2}+M_{3}}{3}$$ (12)

In Eq (12), $Z_{0}^{\left(1\right)}$, $Z_{0}^{\left(2\right)}$, and $Z_{0}^{\left(3\right)}$ represent the initial values, with the subscripts indicating the year to be used. *M*_1_, *M*_2_, and *M*_3_ represent the annual logistics transportation volume for the first three years, with units of 10000 tons. When more time series are used, weights are assigned in chronological order, as calculated in Eq (13).


$$\{\begin{array}{l} Z_{t}^{\left(1\right)}=\phi X_{\left(t\right)}+(1-\phi )Z_{t-1}^{\left(1\right)}\\ Z_{t}^{\left(2\right)}=\phi Z_{t}^{\left(1\right)}+(1-\phi )Z_{t-1}^{\left(2\right)}\\ Z_{t}^{\left(3\right)}=\phi Z_{t}^{\left(2\right)}+(1-\phi )Z_{t-1}^{\left(3\right)} \end{array}$$ (13)

In Eq (13), *t* represents the year. *ϕ* is the optimal smoothing coefficient, and its value is determined to be 0.138 through mean square error calculation. After determining the prediction period and base year, the mathematical model of the cubic exponential smoothing method is represented by Eq (14).


$${\hat{Y}}_{t+T}=a_{t}+b_{t}\times T+c_{t}\times T^{2}$$ (14)

In Eq (14), ${\hat{Y}}_{t+T}$ represents the indicator predicted value with a prediction period of *T* and the *t*th year as the base year. *a*_*t*_, *b*_*t*_, and *c*_*t*_ are smoothing coefficients. The smoothing coefficient is represented by Eq (15).


$$\{\begin{array}{c} a_{t}=3\times (Z_{t}^{\left(1\right)}-Z_{t}^{\left(2\right)})+Z_{t}^{\left(3\right)}\\ b_{t}=\frac{\phi }{2{\left(1-\phi \right)}^{2}}\times [(6-5\phi )Z_{t}^{\left(1\right)}-2(5-4\phi )Z_{t}^{\left(2\right)}+(4-3\phi )Z_{t}^{\left(3\right)}]\\ c_{t}=\frac{\phi^{2}}{2{\left(1-\phi \right)}^{2}}\times [Z_{t}^{\left(1\right)}-2Z_{t}^{\left(2\right)}+Z_{t}^{\left(3\right)}] \end{array}$$ (15)

In Eq (15), *ϕ* represents the optimal smoothing coefficient with a small deviation from the actual value calculated by mean square error, and its range is [0.1,0.9]. By establishing an indicator system for logistics transportation volume, the logistics industry’s transportation volume can predict transportation data for the next five years and provide reference for SLD’s strategic layout and logistics management.

## 4. Results

Through the analysis of enterprise situation and the construction of strategic key factors through SLD, an indicator system analysis was conducted on the four judgment matrices of strategic key factors. SLD and strategy selection were proposed.

### 4.1 Indicator system for sustainable logistics development strategy

**Fig 5:**
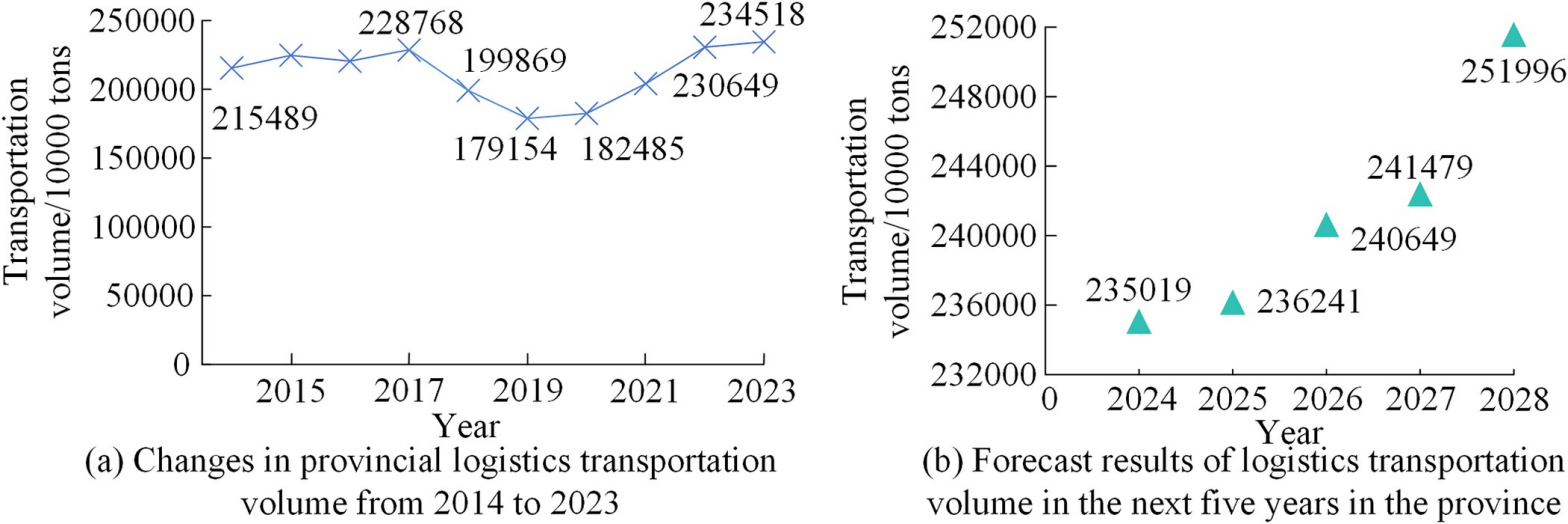
Prediction results of logistics transportation volume smoothing index.

Based on the current situation of logistics transportation and the exponential smoothing prediction of transportation volume, the trend of logistics transportation volume changes in the next five years was analyzed in Fig 5.

From Fig 5A, logistics companies achieved the lowest logistics transportation volume in 2019 and 2020, with 179.154 million tons and 1824.85 million tons, respectively. After 2021, the total logistics transportation continued to rise, reaching a transportation volume of 2345.18 million tons in 2023. In Fig 5B, the logistics transportation volume in 2026 increased by 1.87% compared to 2025, with the fastest change in transportation volume from 2026 to 2027, an increase of 4.36%. The judgment matrix and factor weights of strategic key factors were calculated and consistency checked. Table 2 shows the normalized eigenvectors and consistency test results for each judgment matrix.

**Table 2:** Characteristic vectors and consistency test results of the judgment matrix for strategic key factors.

Judgment matrix	Maximum eigenvalue	V_i_	CR	Whether the consistency check is passed or not
S	4.1631	(0.0956, 0.4728, 0.2299, 0.1982)	0.0611	Yes
W	4.0228	(0.0904, 0.1509, 0.3205, 0.4058)	0.0085	Yes
O	4.1243	(0.5817, 0.1827, 0.0616, 0.1396)	0.0466	Yes
T	4.1251	(0.5525, 0.2477, 0.0636, 0.1018)	0.0707	Yes

**Fig 6:**
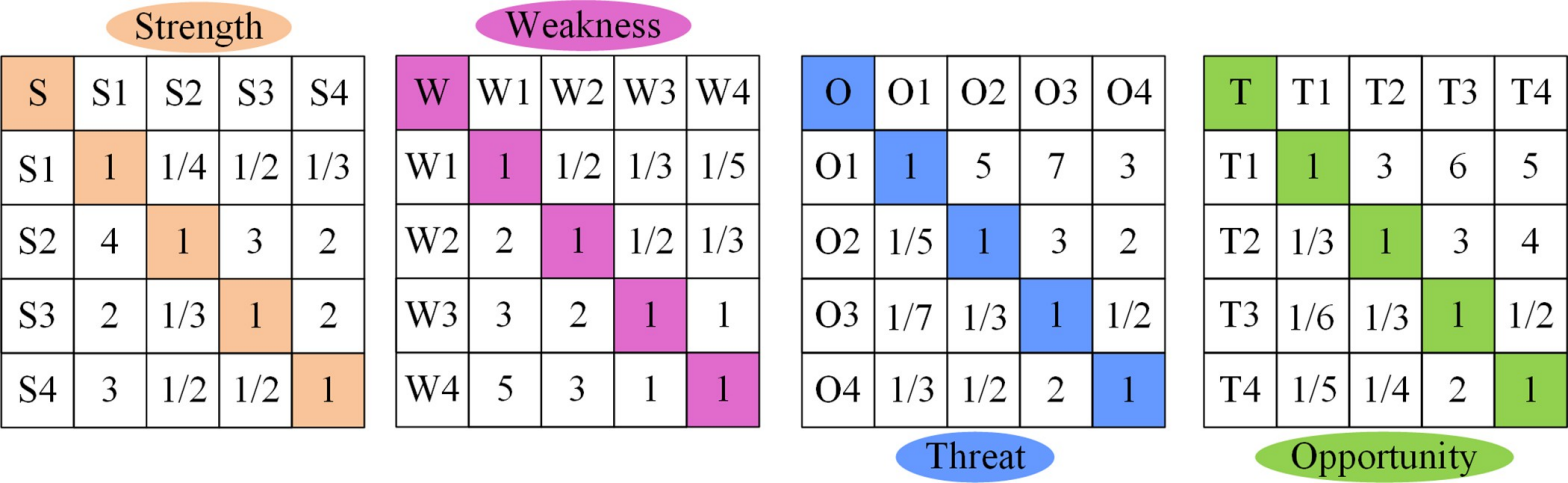
Schematic diagram of the judgment matrix for pairwise factor comparison.

From Table 2, the CR of all four judgment matrices was below 0.5, indicating that they passed the consistency test. This indicated that the development strategy factors combining SWOT analysis and AHP analysis were effective and feasible. Afterwards, the specific factors were added and hierarchically merged into the final weight determined by the overall goal. The indicator system of the factor with the highest weight was the optimal strategic plan. Based on the construction of the SWOT quantitative model and the evaluation of expert questionnaire surveys, a pairwise comparison of strategic key factors in the judgment matrix was conducted in Fig 6. The questionnaire recruitment period was from May 21 2023-June 7 2023.

**Fig 7:**
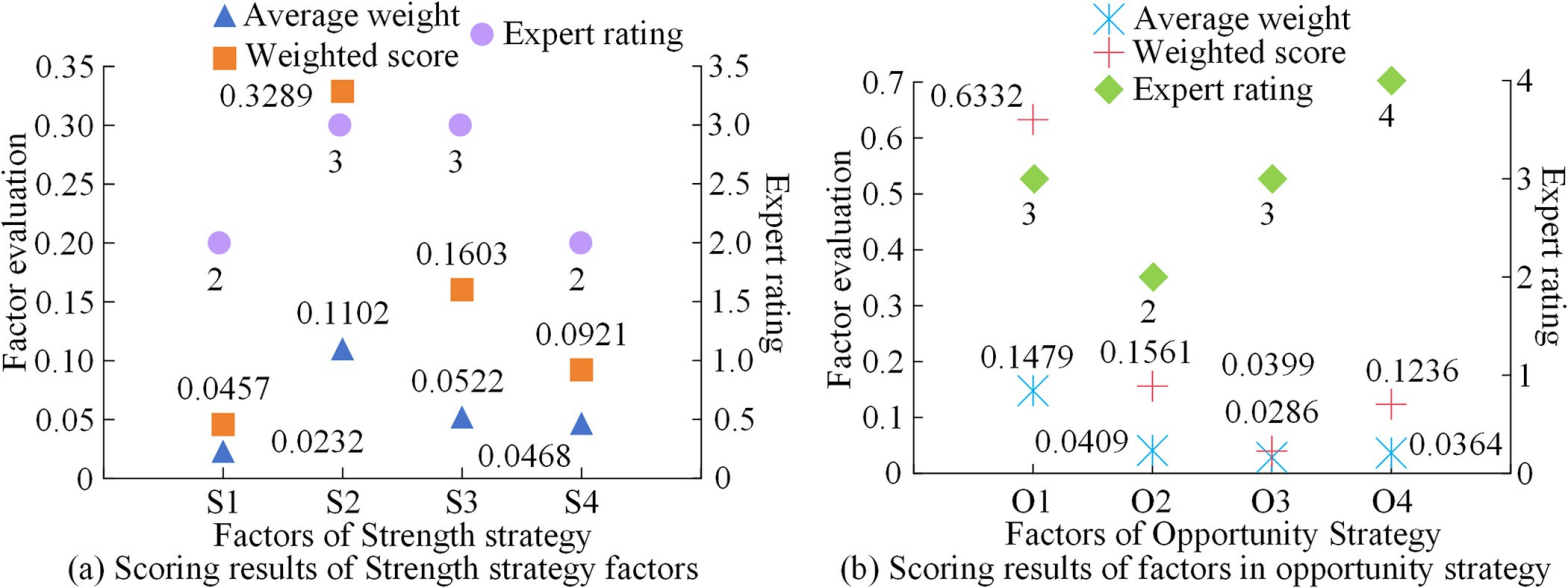
Scoring results of positive intensity strategy factors.

From Fig 6, there were pairwise matrices of four strategic key factors. The specific evaluation factors for their own indicators were divided into 1. However, through the interaction of other indicators, the evaluation result of S1 was good, indicating that the influence of logistics geographic location had a significant advantage on logistics SD. As for the disadvantage factors, industrial structure, and advanced information technology, there was a lack of them. Therefore, modern advanced information technology was needed in SLD to provide weighted results for the industrial structure and its future development direction. Based on the consistency test results of the judgment matrix, the changes in the evaluation and weighted scores of the four strategic key factors were as follows. Fig 7 shows the factor evaluation results of the positive intensity strategy.

**Fig 8:**
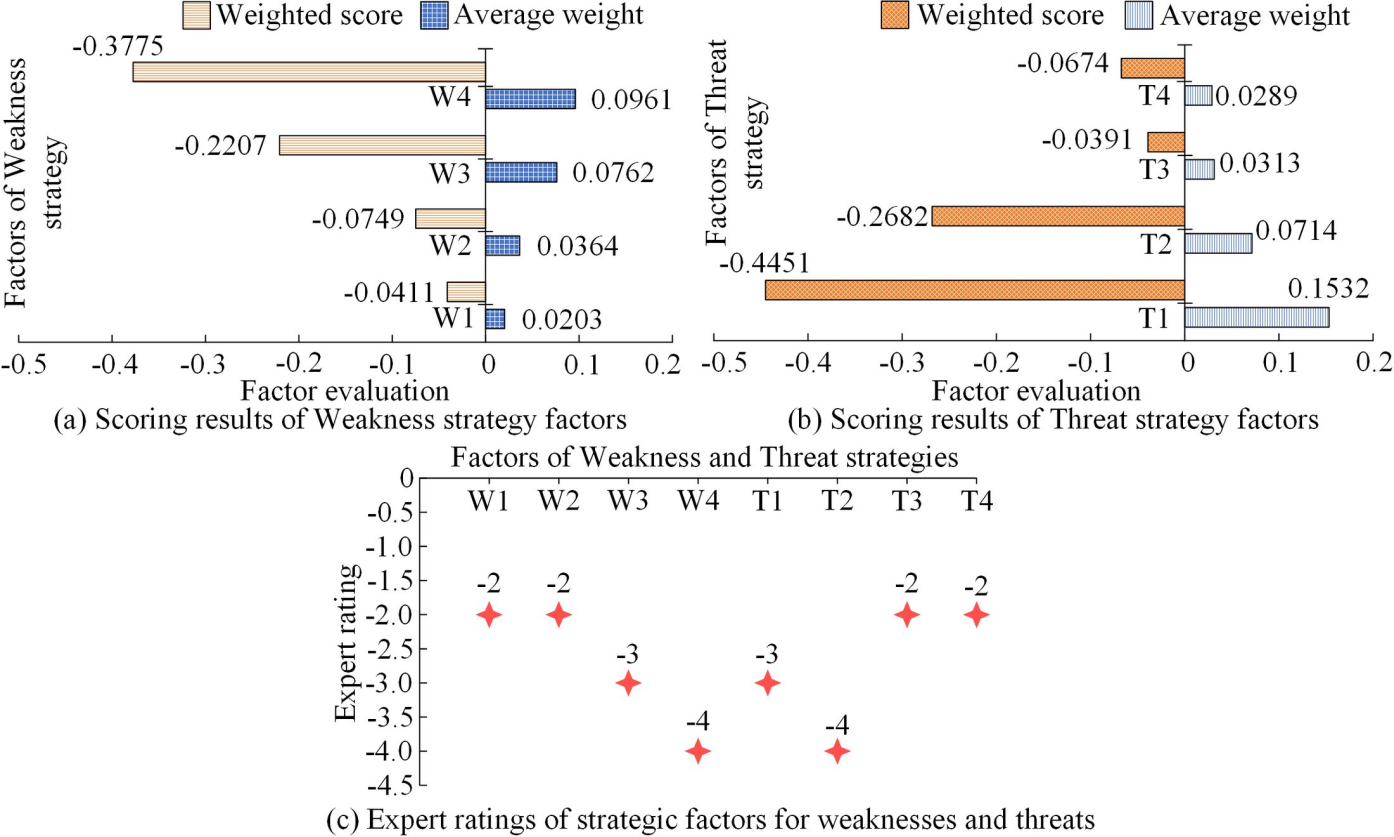
Scoring results of negative intensity strategic factors.

In Fig 7A, the average weights of factors in the advantage strategy of logistics enterprise SD were 0.0232, 0.1102, 0.0522, and 0.0468, respectively. This indicated that the advantages of industrial resources in the logistics industry were more prominent. The expert ratings for green transportation networks were also relatively good. In Fig 7B, the overall evaluation of the factors in the opportunity strategy was good, with an average expert rating of 3. The result of the country’s emphasis on logistics development was 0.1479. The average weights of factors driven by trade development, world logistics trends, and leading enterprises were 0.0409, 0.0286, and 0.0364, respectively. This indicated that external policies and economic opportunities for logistics enterprises and industries were favorable, which could promote the rapid development of logistics enterprises. Afterwards, the evaluation results of negative intensity factors were analyzed in Fig 8.

In Fig 8A, the average weight and weighted score of the factors related to green consumption awareness in the disadvantaged strategy were 0.0961 and -0.3775, respectively. The industrial structure, advanced information technology, and government supervision were also at a disadvantage in the competitive development of enterprises. The required weighted scores were -0.0411, -0.0749, and -0.2207, respectively. In Fig 8B, the four external threat factors faced by the enterprise had mean weights of 0.1532, 0.0714, 0.0313, and 0.0289, respectively, with weighted scores of -0.4451, -0.2682, -0.0391, and -0.0674. Through the analysis of the above indicator system and weighting results, the development of the logistics industry and enterprises is influenced by multiple factors, and these factors are interrelated and interact with each other. Research on the use of strategic decision-making and analysis methods to simplify factor problems and transform them into qualitative mathematical problems. Therefore, based on the weighting results, reliable mathematical analysis can be provided for the strategic types and development directions of different factors, reflecting the importance of factors for development strategies and promoting enterprise target decision-making.

### 4.2 Comparative analysis of sustainable logistics development models

**Fig 9:**
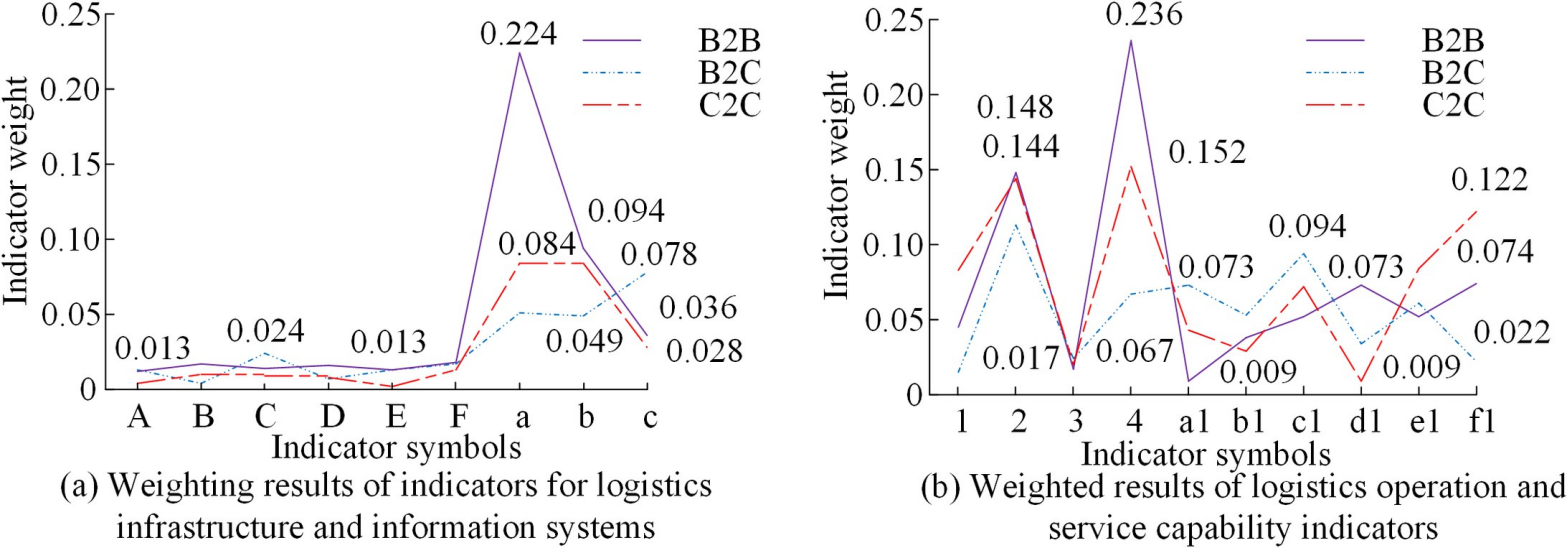
Indicator weight results for three logistics development models.

The information management in the logistics industry tends to be complex. Therefore, the improvement of information technology could promote the SD of the logistics industry and provide a development model for the corresponding industrial structure. As a product of the progress and development of the Internet, e-commerce mainly included the development models of Business to Business (B2B), Business to Customer (B2C), and Consumer to Consumer (C2C). The key factors of SLD were utilized to evaluate the logistics indicators of three development models. Firstly, the indicators of logistics infrastructure mainly include warehousing equipment, transportation, information, network density, facility utilization rate, and per capita transportation volume, which are set as A-F. The information system capability indicators were system assurance capability, direct and indirect economic benefits, which were represented by a-c. Secondly, logistics management capabilities were divided into professional personnel, logistics costs, per capita training, and market share, expressed as 1–4. The final logistics service capabilities included order response, on-time delivery, delivery flexibility, delay rate, error rate, and cost-benefit, which represented by a1-f1 in Fig 9.

**Fig 10:**
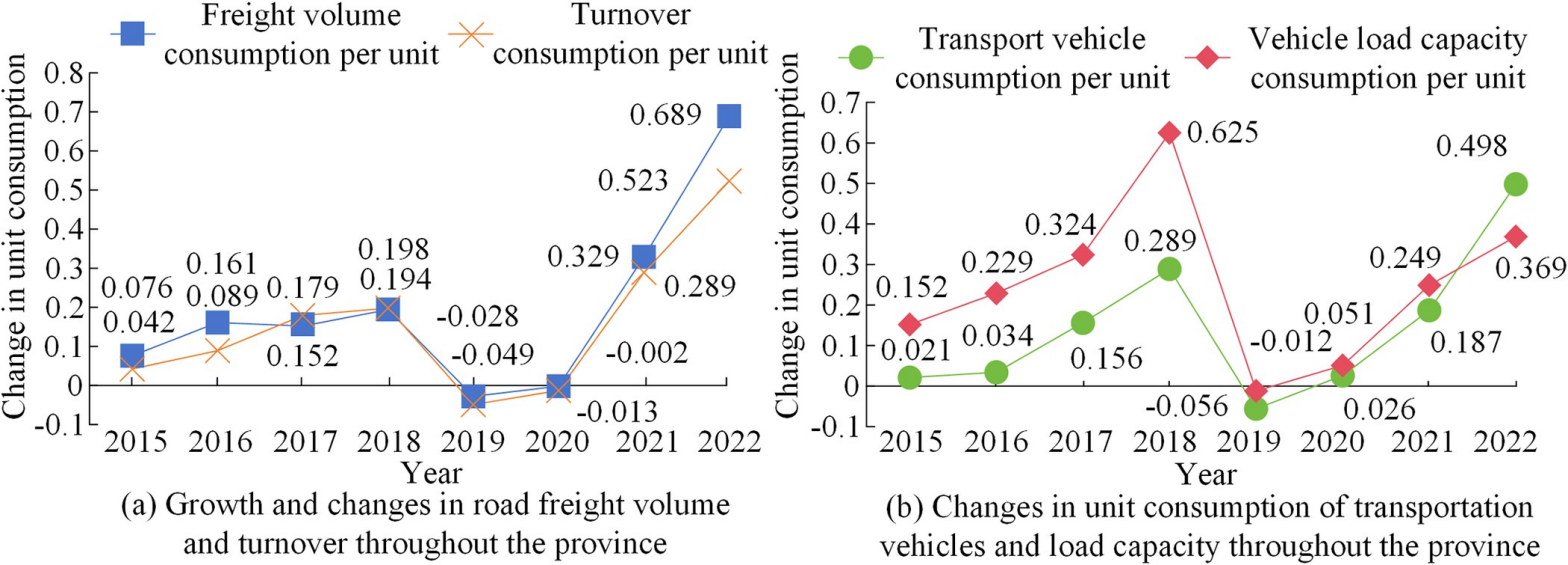
Energy consumption changes of road transportation and vehicles in the province.

From Fig 9A, the weight values of these three modes on logistics infrastructure indicators were relatively low, generally below 0.03. The weight of B2B indicators for information equipment was 0.024. The weight of indicators for warehousing equipment was 0.013. C2C had the lowest weight on the utilization rate of warehousing equipment and logistics infrastructure, with values of 0.004 and 0.002, respectively. In Fig 9B, the logistics cost weight of B2B in logistics management capability was the highest at 0.148. The weight of per capita training expenditure for the three modes was relatively small, with values of 0.017, 0.024, and 0.020, respectively. This study analyzed transportation, packaging materials, and logistics services in the logistics system to provide improvement solutions for the logistics system. The specific indicators of energy consumption for a large amount of transportation workload included the province’s road freight volume, freight turnover, and the unit consumption of transportation vehicles and their load capacity in Fig 10.

**Fig 11:**
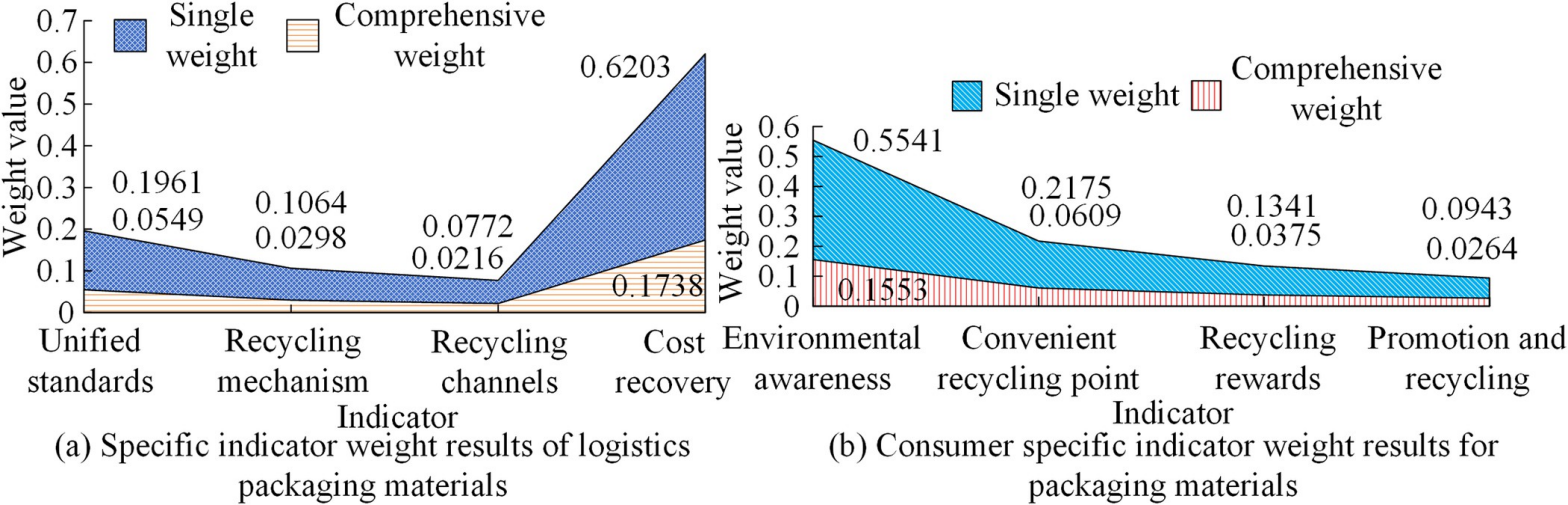
Weighted results of indicators for the recycling of logistics packaging materials.

From Fig 10A, the unit consumption growth of logistics freight volume in the province showed a slow growth before 2018. The unit consumption of freight volume decreased from 2019 to 2020, reaching -2.8% and -0.2% respectively. Subsequently, with the recovery of the social economy, the unit consumption of freight volume increased rapidly, increasing by 68.9% in 2022. The unit consumption of freight turnover increased by 52.3% by 2022. In Fig 10B, there was a significant change in the unit consumption of transportation vehicles throughout the province. The unit consumption of transportation vehicles in 2022 was lower than in 2018, with a value of 36.9%. The unit consumption change of vehicle load was -5.6% in 2019, with the highest value being 49.8% in 2022. However, regarding the recycling of logistics and express packaging materials, the main indicators were logistics and express enterprises and consumers. Their weights were calculated using AHP in Fig 11.

From Fig 11A, the main indicator of logistics companies for packaging materials was the cost of material recycling. The individual weight and comprehensive weight were 0.6203 and 0.1738, respectively. However, there was relatively little development of recycling channels for packaging materials, with the lowest individual and comprehensive weights of 0.0772 and 0.0216, respectively. Therefore, enterprises needed to vigorously increase recycling points and channels for the recycling of packaging materials to save resources and promote green development. In Fig 11B, there were also significant differences in consumer environmental awareness and utilization of packaging materials. Consumers had a good awareness of the recycling of packaging materials, with weight values of 0.5541 and 0.1553, respectively. Finally, through SWOT analysis of logistics enterprises and evaluation of various indicators at all levels, the service quality evaluation of logistics development was summarized in different dimensions to summarize the SLD plan in Table 3.

**Table 3:** Scoring results of sustainable logistics service quality.

Dimension	Specific indicators	Indicator weight	Questionnaire score	Comprehensive score
Environmental dimension	Energy conservation and emission reduction	0.376	3.773	1.419
	Green packaging	0.624	3.189	1.989
Social dimension	Number and time of delivery points	0.473	3.802	1.798
	Green logistics services	0.527	3.761	1.982
Economic dimension	Express delivery price	0.613	3.672	2.251
	Return and exchange fees	0.387	3.524	1.364

According to Table 3, the comprehensive scores of each dimension in the service quality of green logistics were relatively good. The price level score for the economic dimension was 2.251. However, the low score of return and exchange fees indicated that the economic dimension of logistics service points still needed to be reasonably regulated. The scores for express delivery points and green logistics services in the social dimension were relatively high, at 1.798 and 1.982, respectively, indicating an improvement in the service quality of green logistics. In the environmental dimension, the score for green packaging was relatively high at 1.989, indicating that the resource utilization of green packaging had a promoting effect on SLD. Taking into account all the above indicators, SLD strengthened industrial management and institutional improvement in both disadvantage and opportunity factors. Meanwhile, it continuously enhanced advantageous factors to cope with threats and challenges, thereby improving the green logistics services.

## 5. Discussion

This study analyzes the strength crisis of logistics enterprises in terms of green SD in the logistics industry and building an environmentally friendly society. The current situation of enterprise development structure and impact indicators is discussed. By constructing a SWOT quantitative analysis model to extract key factors of development strategy, and combining AHP with specific indicators of key factors, weight calculation and consistency testing were carried out. The transportation link in the logistics system had the greatest impact on resource consumption and ecological environment balance. Therefore, exponential smoothing prediction was used to calculate the changes in transportation volume, thereby determining the green direction of logistics transportation development mode. Yuan et al. [22] used genetic algorithms and weight loading methods to evaluate the transportation loading of their logistics scheduling. The average percentage deviation obtained was 3.96%. After comparison, the highest value-added in calculating and predicting the transportation volume of logistics enterprises in this study was 4.36%. This was because the exponential smoothing prediction method considers the loading and scheduling aspects of logistics transportation, thereby improving the efficiency of logistics transportation and reducing transportation energy consumption. In summary, the analysis of advantages and disadvantages and expert ratings used can clarify the SD direction of logistics. This provided an effective data foundation for the development strategy factors in the evaluation and analysis of the indicator system to accurately and completely understand the development model of logistics enterprises. This could accurately and effectively improve the development strategy and service quality of the logistics industry. Moreover, Solangi et al. [10] employed the SWOT method, AHP method, and fuzzy ranking method in their investigation of sustainable energy planning strategies. This was done with the objective of conducting a reasonable evaluation of the utilization of energy planning in Pakistan, with the intention of achieving sustainable energy development and enhancing the country’s energy security. In comparison, an examination of the SWOT and AHP methods employed by sustainable logistics enterprises could facilitate the strategic analysis required for green development. This was due to the key extraction and weight calculation of development factors by SWOT and AHP methods, which could quantitatively analyze the consistency test of their impact indicators and reflect the SD capability and direction of the industry.

At present, research on technological improvement and management strengthening in the logistics industry mainly relies on computer technology and industrial machinery to achieve automation and intelligence of logistics operations. Through logistics services and system construction in different fields, modern logistics enterprises not only face market competition and industrial technology upgrading, but also carry out marketing operations on their own service levels and development models. This study conducts indicator analysis on the development status of logistics enterprises and industries, and combines comparative analysis of indicator elements to provide data reference for the logistics service system. However, the SWOT method used by the study to delineate and model the factors affecting SLD, as well as the expert assessment opinions, are to some extent subjective. This leads to a lack of dimension in the development strategies of individual logistics companies. The quantification of indicators and the lack of comprehensive data in AHP analysis and questionnaire surveys. Accordingly, the formulation of future research development strategies must consider quantitative indicators of influencing factors from multiple perspectives, accurately reflect the strategic types and capabilities of green logistics development in qualitative and quantitative analysis, and provide a high-quality platform for the sustainable and healthy development model of the logistics industry in the future.

## 6. Conclusion

A logistics development strategy based on SWOT and AHP was proposed for the SLD problem. Firstly, a quantitative model was constructed for four strategic factors using the competitive situation of logistics enterprises. Secondly, a qualitative analysis of the indicator system for strategic factors was conducted using AHP. Then, combining the judgment matrix calculation of the indicator system and the smooth prediction of the logistics transportation index, four levels of analysis were conducted on the current development of logistics enterprises. The fastest growth rate of transportation volume for logistics enterprises in the future was 3.46%. The highest weighted score in SD advantage strategy was 0.3289 for industrial high-quality resources. The weight that China attached great importance to in the opportunity strategy was 0.1479. Finally, a comparative analysis of different indicators was conducted on the relevant logistics development models. The weight of B2B indicators for information equipment was 0.024. The on-time delivery weight for B2C was 0.094. The cost-benefit weight of C2C was 0.122. The unit consumption of logistics freight volume in the province increased rapidly by 68.9% in 2022. The unit consumption of freight turnover also increased by 52.3%. The unit consumption growth of transportation vehicles and vehicle load was the highest at 62.5% and 49.8%, respectively. Consequently, SLD effectively enhances the quality of logistics services, not only increasing the economic and environmental benefits of logistics enterprises, but also strengthening consumer awareness of green environmental protection and promoting the comprehensive competitiveness of logistics enterprises in SD. In the process of formulating SD strategies in the logistics industry and enterprises, relevant entities should leverage their own advantages, overcome disadvantages, seize opportunities, and comprehensively respond to the high-quality development of the logistics industry. This should be done in a way that promotes the construction of green logistics infrastructure and green transportation methods.

However, research on specific operational aspects of logistics systems and information technology still lacks weighting indicators and technological developments. Regarding the use of decision analysis methods, future research could also use the Complex Proportionality Assessment (COPRAS) method. This method not only considers the effects of multiple decision factors, but also compares decision options to ensure their performance under different conditions. There is also the Weighted Aggregated Sum Product Assessment (WASPAS) method, which prioritizes various strategic objectives based on SWOT and AHP methods to improve the rationality and feasibility of enterprise development strategies.
